# Multi-Faceted Assessment of Children with Selective Mutism: Challenges and Practical Suggestions

**DOI:** 10.3390/bs15040472

**Published:** 2025-04-05

**Authors:** Maayan Shorer

**Affiliations:** 1 Clinical Psychology Department, Ruppin Academic Center, Emek-Hefer 4025000, Israel; maayans@ruppin.ac.il; Tel.: +972-523-402-547; 2 The Selective Mutism Unit, Psychological Medicine Department, Schneider Children’s Medical Center of Israel, Petach Tikva 49420235, Israel

**Keywords:** selective mutism, psychological assessment, neuropsychological assessment

## Abstract

The multi-faceted nature of Selective Mutism (SM), and its comorbidity with other disorders, necessitates a comprehensive assessment process. However, evaluating children with SM presents significant challenges, including difficulties in building rapport, establishing an accurate diagnosis, and conducting formal psychological and neuropsychological assessments. This paper explores the key obstacles in assessing children with SM and provides practical recommendations for overcoming these challenges. Effective strategies for reducing anxiety during assessments include extended rapport-building phases, playful and engaging interactions, and the strategic use of parental involvement. Additionally, given the variability in SM symptoms across different settings, a multi-informant and multi-method assessment approach—including clinical observation, structured interviews, and standardized parent- and teacher-report measures—is recommended. This paper also discusses adaptations for formal testing, particularly in cognitive, language, and neurodevelopmental assessments, where SM-related speech avoidance can interfere with standardized evaluations. Nonverbal assessment tools, modifications to testing environments, and alternative response formats are proposed as potential solutions. Furthermore, we highlight the importance of differentiating SM from overlapping conditions, such as autism spectrum disorder and language impairments, to ensure accurate diagnosis and intervention planning. By implementing tailored assessment strategies, clinicians and researchers can improve diagnostic accuracy and better understand the unique needs of children with SM. This, in turn, can inform individualized treatment plans, enhance educational placement decisions, and support the overall well-being of children with SM.

## 1. Challenges in the Assessment of Children with SM

Selective Mutism is a relatively rare anxiety disorder, which tends to coexist with other anxiety disorders, with specific behavioral and temperamental characteristics, and with other neurodevelopmental difficulties (Muris & Ollendick, 2021). The multi-faceted nature of this disorder often requires a thorough assessment process. However, oftentimes this process encounters significant challenges and obstacles. The current paper aims to describe the different aspects of the assessment process with children with SM, present the challenges it meets, and suggest practical ways to minimize or overcome them.

The diagnosis of SM is given when the child fails to speak in certain social situations (e.g., at school), despite having the ability to speak regularly in other situations (e.g., with close family members), and when the failure to speak interferes with the child’s achievements or social functioning (American Psychiatric Association—APA, 2022). SM is categorized as an anxiety disorder in the latest edition of the Diagnostic and Statistical Manual of Mental Disorders (DSM V-TR; APA, 2022) and has very high rates of comorbidity with other anxiety disorders (Driessen et al., 2020; Muris & Ollendick, 2021; Vecchio and Kearney, 2005).

SM involves various difficulties beyond failure to speak (Vogel et al., 2024). Children with SM typically tend to be behaviorally inhibited and thus prefer avoiding novel or unfamiliar situations, interpersonal interactions, and other stimuli (Gensthaler et al., 2016). They also tend to exhibit freezing responses when feeling threatened, which can be observed in reduced motor activity (including gestures) and the inability to vocalize (Vogel et al., 2019; Vogel et al., 2022).

Given these characteristics, the assessment of children with SM may encounter different challenges, starting with difficulties in building rapport, followed by difficulties in establishing a diagnosis of SM and differential diagnosis and achieving cooperation in formal tests. In the following sections, we will describe some of these challenges and offer practical means to handle them.

## 2. Challenges and Practical Suggestions for Building Rapport and Minimizing Anxiety During the Assessment Process

Children with SM tend to be inhibited in new and unfamiliar situations (Gensthaler et al., 2016). They may freeze at the start of the assessment process or try to avoid it. Often, these children need more time to feel comfortable in the evaluation room and with the evaluator’s presence. Therefore, a longer time should be allocated to decreasing anxiety, habituating to the new environment, and building rapport with the evaluator. When clinicians are prepared in advance to evaluate a child with suspected SM, they can plan the evaluation process such that it starts with a session devoted only to these purposes. In this session, the child can have free play with his/her parent in the evaluation room. The evaluator can start by watching them from the side and then gradually “slide in” and join the play (Hung et al., 2012). An unprepared examiner who encounters a child with suspected SM can slow down the evaluation process and allow more time for a “warm-out”. The importance of this “warm out” phase was demonstrated by Kurtz and Comer (2011), who reported the response rate of children with SM who had a brief rapport-building segment with an unknown examiner. A total of 27% of the children verbally responded to the first question asked by the examiner, 36% responded to the second question, and 43% answered when the third question was posed. Engaging in play and encouraging a sense of playfulness and humor are often effective ways to help reduce anxiety in children (Weisman & Shorer, 2017). We recommend that the evaluator create an informal, pleasant, and playful atmosphere.

Initial attempts to verbally communicate with the child can be made when the child appears more relaxed and take place within the context of play (Bergman, 2013). For example, the evaluator can introduce simple choice questions to the child as part of a “21 Questions” game. In this game, one player chooses an object, a famous character, a movie, etc., and the other player must guess it by asking up to 21 yes/no questions. Additionally, we recommend that in this initial phase, the evaluator should limit verbal communication with the child to closed-ended questions or simple questions that the child is likely to know the answers to (e.g., “How many brothers and sisters do you have?”). It is preferable to ask questions that require a one-word oral response, rather than those that can be answered by physical gestures (such as nodding or hand signing), unless the child appears unable to provide such oral responses. Questions that require the child to choose between two or three options can be helpful (e.g., “I hear you like to watch the sports channel. Do you prefer football or basketball?”). Choice questions can lessen the burden on the child, as they require recognition instead of recall (which can be more intimidating for children with SM).

As children become more comfortable, the evaluators can gradually increase their verbal communication with the child (Hung et al., 2012). However, in our experience, open-ended questions and inquiries about personal or subjective matters can often be intimidating for children with SM.

Contingency management techniques can be employed to encourage the child to engage in conversation with the evaluator. For example, the child could earn stickers for each attempt to speak. It is crucial for the evaluator to avoid giving ultimatums to the child when failing to respond. Instead, a validating and encouraging approach is recommended. This means acknowledging the child’s difficulty in speaking while also expressing confidence in their ability to make progress. Limited eye contact can also be beneficial in reducing a child’s anxiety (Bergman, 2013; Oerbeck et al., 2014). For instance, evaluators can sit with their back turned to the child, communicate while facing the computer screen, or even ask a question and step out of the room for a few minutes, allowing the child to respond more comfortably to the parent. The parent’s presence during this phase (and, in many cases, throughout the entire assessment process) is important for the child’s feelings of safety and for aiding communication with the child. Parental presence was found to help in reducing children’s anxiety in medical settings and procedures (Sadeghi et al., 2016; Rastegarian et al., 2016).

## 3. Challenges and Practical Suggestions for Establishing a Diagnosis of SM

SM is a multi-faceted disorder, which means that the evaluation process of these children must be comprehensive and multi-informant based (Viana et al., 2009). As SM, in its nature, is a condition characterized by varying levels of functioning in different environments, gathering information from multiple sources is particularly important. Parents’ interviews, clinical observation, and information from teachers are all advised in this process (Dow et al., 1995). It should be noted that the children’s verbal communication with the clinician, or in the clinical setting, does not necessarily correlate with their speaking in everyday situations (Black & Uhde, 1995). A child with SM may speak fluently with the clinician conducting the assessment but consistently avoid speaking to their teacher. Therefore, a normal performance in one setting should not rule out a diagnosis of SM, and a comprehensive view of the child’s speech across different environments should be achieved.

Using a variety of information sources can also aid in making a differential diagnosis. The DSM-V-TR (APA, 2022) specifies that a person’s inability to speak in certain social situations should not be attributed to a lack of knowledge or understanding of the spoken language required. However, studies have shown that immigration and bilingualism occur at higher rates among children with SM (Slobodin et al., 2024). In such cases, it is important to compare the child’s speech in different settings, where familiar or unfamiliar languages are used (for example, speaking with grandparents versus interacting with peers at school). SM in children learning a second language (e.g., immigrants, bilingual) can be suspected when the mutism is prolonged, not in line with the child’s understanding of the second language, and present in both languages (typically with more pervasive avoidance of the second language). For a more detailed discussion, refer to Toppelberg et al. (2005).

The significance of gathering information from multiple sources is also evident when trying to differentiate between SM and autism spectrum disorders (ASD; Oerbeck et al., 2019). While children with SM exhibit speech challenges in specific contexts, those with ASD generally experience communication challenges across a wider range of situations. Therefore, it is essential to evaluate a child’s speech, as well as broader developmental aspects (e.g., language, motor, social skills), with different individuals and in various settings. A useful method to differentiate SM from ASD is to videotape the child in a situation that does not elicit anxiety, such as while interacting with a familiar person in a familiar surrounding and absorbed in a playful or joyful activity.

Another challenge in determining a diagnosis of SM is that its definition is somewhat vague (Oerbeck et al., 2019). For instance, it is unclear whether a child who only whispers when talking to adults, or a child who rarely answers the teacher’s questions, meets the DSM-V-TR (APA, 2022) criteria for a consistent failure to speak in specific settings. One effective way to support the diagnosis of SM is by using structured, reliable, and validated assessment tools (Rodrigues Pereira et al., 2023). The Anxiety Disorders Interview Schedule for Children and Parents (ADIS-C/P: Silverman & Albano, 2024) is a semi-structured interview, delivered by clinicians to assess different anxiety disorders, and SM is among them. It provides both threshold criteria for diagnosis and a severity scale. Another option is to use parents’ reports on validated questionnaires. The Selective Mutism Questionnaire (SMQ; Bergman et al., 2008) is a measure with good psychometric properties designed to assess the level of SM in different settings, using parents’ reports. This scale consists of 17 items measuring the frequency of children’s non-speaking behavior at home, in school, and in other public/social situations. Another instrument is the Frankfurt Scale of Selective Mutism (FSSM; Gensthaler et al., 2020), a parent-report measure that includes both a diagnostic scale to assist in establishing a diagnosis of SM, and a severity index of SM symptoms. The FSSM has three age versions: 3–7, 6–11, and 12–18 years. The FSSM also provides an optimal cutoff score to differentiate children with SM from children diagnosed with social phobia, other internalizing disorders, and a non-clinical group. This questionnaire is freely available online in several languages. The parents’ reports can be supplemented by teachers’ reports, such as The School Speech Questionnaire (SSQ; Bergman et al., 2002), which is a teacher-based measure that assesses the frequency of children’s non-speaking behavior at school. A comprehensive and updated critical review of measures assessing SM can be found in Rodrigues Pereira et al. (2023).

Behavioral observations, in both naturalistic and non-naturalistic settings, may also support the process of establishing an SM diagnosis. The clinician may observe the child’s speaking behaviors by visiting the child’s school, or by watching videotapes filmed by the parents. These naturalistic observations require clinicians to consider ethical issues, such as verifying the child’s informed consent, particularly with older children. In our clinical experience, observing the child–parent interaction in a non-naturalistic setting (e.g., clinical setting)—either directly or through a one-way mirror—can provide valuable insights into the child’s performance in unfamiliar contexts. We typically set up a room equipped with various toys, games, and craft materials, allowing children to choose their preferred activities. This observation opportunity enables us to gain an impression of the child’s linguistic and social communication abilities, temperament, and the dynamics of the child–parent relationship.

Bergman et al. (2013) suggest a structured observation of the child’s speech behaviors, using verbal and nonverbal interactional tasks. Examples of nonverbal tasks include activities like blowing bubbles, jumping up and down, and posing for an instant photo. Verbal tasks involve the child responding to a series of neutral questions, such as, “What is your brother’s or sister’s name?” and “What is your favorite color?” Overall, this structured procedure contains 10 items with a total duration of 10 to 15 min. A similar observational procedure, which also includes a coding scale, is described by Shorer et al. (2023). Another structured observation for SM was described by Milic et al. (2020). This procedure assessed the child’s verbal and nonverbal functioning during waiting time, nonverbal social interaction with an unfamiliar assessor, and verbal social interaction with the unfamiliar assessor. The latency, number, and duration of verbal/nonverbal gestures are all assessed.

Behavioral observations may also be used to distinguish SM from ASD in children who do not speak in specific social situations. Muris & Ollendick (2021) propose a two-step approach to assessing ASD symptoms. The first step involves using designated screening tools for ASD, such as the Social Responsiveness Scale—second edition (SRS-2; Constantino & Gruber, 2012). The second step consists of a standardized observational procedure, such as the Autism Diagnostic Observation Scale (ADOS-2; Lord et al., 2012). However, it is important to note that the existing screening tools, observations, and interviews for ASD (e.g., SRS-2, ADOS-2) were shown to have insufficient ability to discriminate ASD from SM and other anxiety disorders (Capriola-Hall et al., 2021; Cholemkery et al., 2014; Wittkopf et al., 2022). This limitation often leads to a high rate of false positives. Therefore, it is strongly recommended to interpret their results with caution and to conduct a thorough clinical assessment of the child’s functioning across a broad range of situations.

## 4. Challenges in Using Formal Tests with Children with SM

### 4.1. Assessing Comorbid Neurodevelopmental and Language Difficulties

Neuropsychological assessment may be necessary for some children with SM, as neurodevelopmental factors are involved in a significant proportion of them (Muris & Ollendick, 2021). Research shows that children with SM exhibit more delays in both fine and gross motor skills, along with increased rates of neurodevelopmental challenges. These challenges may include developmental coordination disorder, enuresis, encopresis, mild intellectual disability, and autism spectrum disorders (Kristensen, 2000). It has been suggested that in addition to the child’s anxiety, neurodevelopmental factors may contribute to speaking avoidance (Muris & Ollendick, 2015).

A high prevalence of speech and language problems has also been found among children with SM (Ford et al., 1998; Kolvin & Fundudis, 1981; Kristensen, 2000; Kristensen & Oerbeck, 2006; Manassis et al., 2007; McInnes et al., 2004; Nowakowski et al., 2009; Steinhausen & Juzi, 1996). It is important to assess speech and language problems when SM is suspected, for several reasons: First, to establish a diagnosis of SM, the clinician should distinguish between language competence and performance (Klein et al., 2013). The DSM-V-TR (APA, 2022) states that the diagnosis of SM should exclude children whose speech avoidance can be better accounted for by a language disorder. At the same time, it is crucial to identify whether speech or language impairments contribute to the child’s speech avoidance (McCormack et al., 2009) and serve as a risk factor that enhances the child’s vulnerability to SM (Sharp et al., 2007). In such cases, speech and language impairments must be treated as part of a holistic treatment plan in SM. In addition, a lack of participation in verbal interpersonal communication may increase gaps in language development and harm the development of age-appropriate communication skills (Klein et al., 2013).

An assessment of academic performance may also be required for some children with SM (Oerbeck et al., 2019). Although there is empirical evidence that the performance of children with SM in math and reading is comparable to non-clinical controls on standardized tests and teachers’ reports (Cunningham et al., 2004), others found that children with SM and children with anxiety disorders scored significantly lower than community controls in mathematics tests (Nowakowski et al., 2009). In addition to these inconclusive findings, it may be suggested that SM might affect children’s academic coping. Speaking avoidance may disrupt children’s participation in the learning process, such as their initiative to ask for clarifications, complete oral assignments, and participate in group conversation during lessons (Nowakowski et al., 2009). These might lead to teachers’ difficulties in estimating the child’s academic skills due to their speaking avoidance (Dow et al., 1995).

Assessing neurodevelopmental, language, and academic difficulties in children with SM can be quite challenging, particularly when formal standardized tests are necessary. The benefits of standardized tests are well established (e.g., Kaplan & Saccuzzo, 2009). By adhering to a standard procedure, a child’s performance can be compared to age-matched norms and standards, providing valuable insights into both typical and atypical performance. However, the implementation of formal assessments in cases of SM may be complicated by the child’s tendency to avoid speaking, exhibit freezing behaviors, or show low cooperation with an unfamiliar examiner. For that reason, the use of formal tests in children with SM remains limited (Cleator & Hand, 2001). Although nonverbal measures are available for some areas of functioning, many of the commonly employed assessment batteries require oral responses (especially given the need to assess language ability). In addition, even when nonverbal tests are available, the children’s elevated anxiety, inhibited psychomotor responses (Milic et al., 2020), the inclination for perfectionism (Vogel et al., 2019), and the frequent use of safety behaviors (such as frequent “I don’t know” answers at the slightest doubt), might affect their performance and outcomes, which may lead to an underestimation of their abilities or lack of information.

### 4.2. Assessing Psychological Aspects in Children with SM

A psychological assessment is sometimes needed for an in-depth understanding of the child’s inner world (e.g., personality structure and traits, subjective experience, hidden conflicts and wishes, etc.). This type of assessment may assist in providing a diagnosis, addressing the child’s emotional difficulties, selecting the type of treatment, supporting court decisions, etc. (Board of Trustees of the Society for Personality Assessment, 2006). Most of the psychological tests, including projective measures, such as story-telling, sentence-completion tasks, or the Rorschach ink-blot test, might be difficult to apply with children with SM, either because it leans on verbal communication, or requires an open, self-disclosing approach, which is an obstacle for children with SM, due to the timid nature of many of them.

Given the obstacles involved in using formal tests with children with SM, some practical suggestions might be considered. The next section will address issues relating to the choice of tests, adaptations in the administration procedures to the special needs of children with SM, and aspects of SM relevant to interpreting the results.

## 5. Practical Suggestions for Using Formal Tests with Children with SM

### 5.1. Choosing Appropriate Tests

Whenever possible, a preference for nonverbal tests, or tests with minimal oral responses, is advised for children with SM (Dow et al., 1995). Such tests can provide valuable information on important cognitive aspects, such as attention span, memory, executive functions, and processing speed (Dow et al., 1995). Language assessment can utilize tools that include auditory comprehension scales. These scales examine a child’s ability to understand spoken language and cover aspects such as semantics, morphology, syntax, integrative language skills, and phonological awareness. This is accomplished by asking the child to point to a picture or follow simple instructions, thus being suitable for a child who avoids speaking in the testing context. Some examples of such tests are the Preschool Language Scale—5 (PLS-5; Zimmerman et al., 2011) and the Peabody Picture Vocabulary Test—5th Edition (PPVT-5; Dunn, 2019). The assessment of expressive language in children with SM is usually more challenging, yet very significant, because such language deficiencies may impact the child’s willingness to speak (such as if the child has speech production errors or stutters). Expressive language assessment in children with SM can rely on tools that require minimal oral responses (Klein et al., 2013). An example is the Expressive Vocabulary Test—Third Edition (EVT-3; Williams, 2018), which assesses expressive vocabulary for single words. At the EVT-3, examinees are shown pictures and asked to label them or provide one-word synonyms for the labeled pictures. Some of the initial tasks of the PLS-5 expressive communication subscale can also be manageable for children with SM. In cases where a child with SM can speak relatively freely with the examiner, a more comprehensive assessment of expressive language can be performed, for example by using the Test of Narrative Language—second edition (TNL-2; Gillam & Pearson, 2017).

It is important to consider that even when nonverbal tests are administered, the performance of children with SM may be adversely affected by their anxiety levels. Therefore, it is recommended to supplement the formal assessment of cognitive, behavioral, and emotional aspects with reports from parents and teachers using validated questionnaires, such as the Behavior Assessment System for Children—Second Edition (BASC-3; Reynolds & Kamphaus, 2015), the Child Behavior Checklist (CBCL; Achenbach, 2001), and Conners 3 (Conners, 2008).

### 5.2. Adapting the Administration of Formal Tests to the Special Needs of Children with SM

In cases where the assessment is aimed at evaluating abilities or mental aspects that cannot be tested by nonverbal means, adaptations of the formal tests would be needed. This might be true, for example, in the assessment of complex language skills (Dow et al., 1995) or in most projective tests (e.g., TAT, HTP, sentence completion). Some researchers found it helpful to test children in their homes so they could feel less anxious (Cleator & Hand, 2001; Kolvin & Fundudis, 1981). Another possible adaptation of tests that require oral responses is to use audio or videotaping of the child speaking (Cleator & Hand, 2001; Dow et al., 1995; Kolvin & Fundudis, 1981). In other cases, a written response can replace the spoken one.

Other researchers have also recruited parents’ assistance in presenting test stimuli to support standardized testing. In the study of Klein et al. (2013) parents of children with SM were trained to administer tests of receptive and expressive language. The parents were monitored during testing by a professional who scored and interpreted the results. This study demonstrated that parents accurately administered the testing materials. It was also found that children with SM performed significantly better when parents supported the testing administration.

The timing for conducting formal testing should also be carefully considered. In some cases, it may be advisable to wait until there is some symptom relief following the start of medication or psychotherapy. If a child is in the early stages of behavioral therapy and is gradually being exposed to speaking with relatively ‘safe’ individuals, having the child speak to an unfamiliar examiner during formal tests might be too overwhelming. Such a ‘leap’ could potentially jeopardize the gentle, gradual exposure approach being used in therapy.

### 5.3. Interpreting Results of Formal Tests

Numerous findings indicate that trait anxiety and a diagnosis of different types of anxiety disorders are related to adverse effects on neuropsychological performance, such as executive function, memory, attention, and learning (Bishop, 2009; Eysenck et al., 2007; Kalanthroff et al., 2016; Tempesta et al., 2013; Owens et al., 2014). Thus, in the case of children with SM, where anxiety is a dominant factor, the child’s strengths and difficulties, as measured by the different performance tests, might be confounded with his/her anxiety and avoidance. Anxiety may also interfere with the child’s performance on psychological tests (Meyer et al., 2011; Slavin-Mulford et al., 2016). The child’s shyness and inhibited speech might be manifested by a very restricted and concrete response style in these tests. Such responses might be interpreted as reflecting a resistant approach to the test, or limited imagination and creativity, instead of anxiety and stress.

One approach to clarifying such confounding cases is comparing the child’s performance in various conditions and different contexts. We propose two comparisons for clarification:

a. Oral vs. non-oral responses, and verbal vs. nonverbal tests. The most straightforward strategy involves comparing a child’s performance in tests that require a spoken response with their performance in tests where they can respond by writing, signing, or using motor gestures. A broader comparison would be between verbal (not necessarily oral) and nonverbal tests. An example is comparing the children’s performance on the Similarities (a verbal test) and the Block Design (a nonverbal test) subtests of the Wechsler Intelligence Scale for Children, Fifth Edition (WISC-V; Wechsler, 2014), to assess their ability to analyze and synthesize information. Higher scores on a nonverbal test (Block Design) compared to the verbal test (Similarities) can suggest that anxiety lowered the child’s performance on the verbal test, and it can also indicate that verbal language is an area in need of intervention.
b. High vs. low anxiety eliciting tests. Another way to elucidate the role of anxiety in the child’s performance is by comparing the child’s scores in tests that elicit anxiety with less stress-inducing tests. While the testing situation itself can induce stress, some tests are more intimidating than others. For instance, tests with time limits, those that have right or wrong answers, and tests that closely resemble school exams can be particularly stressful. In addition, higher anxiety might be evident at the beginning of the assessment process before the child becomes acquainted with the examiner and the new surroundings. We can also compare the child’s assessment results from tests taken at home with those conducted in more formal settings, such as clinics or schools.

## 6. Conclusions

SM is a complex multi-faceted condition that requires a comprehensive and thorough evaluation of different aspects of the child’s functioning. Despite the inherent difficulties in assessing children with SM, and particularly in administrating formal tests, some adaptations of the assessment process may assist in delivering this essential clinical element. These adaptations include special attention to building rapport with the children and decreasing their anxiety, choosing appropriate tests that are sensitive to children’s speech avoidance and high anxiety, flexible administration of the tests, and using multi-informant and multi-method assessments. Such a comprehensive evaluation would enable better treatment planning, providing adequate services and suitable placements in an educational setting, and a deeper understanding of the child’s inner world.

## Data Availability

Data supporting this review can be found by approaching the author.
